# Access to healthcare for the most vulnerable migrants: a humanitarian crisis

**DOI:** 10.1186/s13031-015-0043-8

**Published:** 2015-05-07

**Authors:** Kevin Pottie, Jorge Pedro Martin, Stephen Cornish, Linn Maria Biorklund, Ivan Gayton, Frank Doerner, Fabien Schneider

**Affiliations:** Departments of Family Medicine and Epidemiology and Community Medicine, Scientist, Ottawa Primary Care Research Group, Bruyere Research Institute, University of Ottawa, 75 Bruyere St, K1S 0P6 Ottawa, ON Canada; Medecins Sans Frontieres, Buenos Aires, Argentina; MSF Canada, 720 Spadina Avenue, Suite 402, M5S 2 T9 Toronto, Ontario Canada; Humanitarian Affairs, MSF OCA, Postbus 10014, 1001 EA Amsterdam, The Netherlands; MSF United Kingdom, 67-74 Saffron Hill, EC1N 8Q London, UK; MSF Germany Bank für Sozialwirtschaft Konto: 97 0 97, Poland, Germany

**Keywords:** Undocumented migrants, Asylum seekers, Refugees, Access to care, Humanitarian crises

## Abstract

A series of Médecins Sans Frontières projects for irregular migrants over the past decade have consistently documented high rates of 14 physical and sexual trauma, extortion and mental illness amidst severe healthcare, food, and housing limitations. Complex interventions were needed to begin to address illness and barriers to healthcare and to help restore dignity to the most vulnerable women, children and men. Promising interventions included mobile clinics, use of cultural mediators, coordination with migrant-friendly entities and NGOs and integrating advocacy programs and mental health care with medical services. Ongoing interventions, research and coordination are needed to address this neglected humanitarian crisis.

## Correspondence/findings

Up to 20% of 232 million international migrant women, children and men are vulnerable to illness and death as refugees or undocumented migrants [1]. The war in Syria alone has displaced 7 million persons— more than 3 million are internationally displaced and many have sought temporary shelter in adjacent countries, but many others have fled across international borders and international waters toward Europe. Along with drownings in the Mediterranean and South Asia seas, asylum seekers and undocumented migrants face increased gross insecurity; for example, in Central America violence, drug wars and extortion have become intertwined with migration.

## Environmental and situational challenges

As high income countries attempt to manage unprecedented migration levels, undocumented migrants and asylum seekers often suffer discrimination and lack of access to healthcare. International migration policies seek to control the flow of migrants using patrol boats in international waters, detention centres along borders, third country resettlement, and restrictions in social and health care policies [2]. Walls have been built, borders closed, yet migration continues. Walls paradoxically put people who are migrating in even more precarious living and healthcare situations without security and access to water, food, and shelter [3].

Detention centres and service cuts reduce access to healthcare for migrants [4]. There are critical periods during the migration trajectory when pregnant women, young children, persons with mental health problems and victims of violence face emergent healthcare needs. Murder, kidnapping, extortion, capture and rape drives migrants into hiding or detention in low and middle income countries and transit cities [5,6].

## The lived experience: case accounts

A case that comes to mind is one of a man detained at a border police station in Evros, Greece [4]. The man describes a 64 day detention in a small cell built for 35 people, however, contained 124 others, *“There is no space to lie down. You cannot walk to go to the toilet… We are treated like animals- worse than animals.”* Another case account given by a 46 year old West African woman trapped in a Moroccan corridor highlights the despair and hopelessness, *“They made us lie on the ground …Each one of us was raped by six men. As soon as one finished, another started… My life is over* [7].*”*

## Detention and deterrence programs

Detention centres near international borders function as field prisons, but with inadequate and often unmonitored healthcare. Detainees face severe marginalization often without recourse or consideration of human rights [8]. For example, a Somali held in an Italian-funded detention centre in Libya has little hope of help from Somalia or Libya—which has not signed any of the Geneva human rights conventions, or Italy, who may be paying Libya to detain Somalis. With increasing frequency, the international migration authorities are launching detainment programs with limited healthcare; for example, in Mexico, Morocco, Egypt, Turkey, Croatia, Indonesia, Malaysia, Nauru and Papua New Guinea [4,5,9].

Humanitarian projects have attempted to address gaps in social and healthcare services. In Morocco, nearly half of all Medecins Sans Frontieres (MSF) medical visits (5,233 in 2012) were related to poor social and living conditions due to insecure travel routes and precarious settlements [5]. In Mexico in 2012, MSF provided 5800 medical consultations, 10% (600) were for physical violence. MSF also provided a total of 1000 psychological consultations, 42% were directly related to violence. Eighty percent of migrant patients in Mexico reported witnessing violence. In 2011, MSF field workers in Libya documented lack of healthcare and lack of back social services amongst the undocumented migrants during the Gaddafi regime. Migrants consistently report fear of disclosure of their status as a barrier to seeking help. As a result, these capture, detention and deterrence programs have now led to organized torture and extortion from migrants and their families that are unmonitored by humanitarian organizations [8].

## Lessons learned from field healthcare projects

- Mobile clinics reach migrants who are too afraid or unable to come to stationary medical clinics.
- Cultural mediators improve triage for emotional, physical and emergency medical needs of migrants.
- Collaboration with local migrant-friendly non-governmental organizations improves reach and access to hidden migrant populations.
- Field programs that address mental health and social needs along with medical conditions play an important role in supporting migrants’ dignity.
- Programs focused on the most vulnerable migrant populations and tailored field programs to the local sociocultural context are viewed as most successful [10].
- Migrant healthcare projects need more research and eventually international best practices.

Advocacy is needed to ensure dignity, healthcare and access to essential medications for displaced women, children and men. Enabling dignity may boost psychological resilience [11], which can help migrants survive prolonged harsh conditions. The key to reaching mobile migrants is mobility, and willingness to reimagine current humanitarian approaches. Violence and insecurity represent a new challenge for non-governmental organization monitoring and action. Ongoing interventions, research and humanitarian coordination are needed to ensure ethical and effective healthcare implementation in remote and insecure areas.

## Abbreviation

MSF: Medecins Sans Frontieres
